# Proteome-wide analysis reveals G protein-coupled receptor-like proteins in rice (*Oryza sativa*)

**DOI:** 10.1080/15592324.2024.2365572

**Published:** 2024-06-21

**Authors:** Dinesh K. Yadav, Gyan Prakash Srivastava, Ananya Singh, Madhavi Singh, Neelam Yadav, Narendra Tuteja

**Affiliations:** a Plant Molecular Biology and Genetic Engineering Laboratory, Department of Botany, University of Allahabad, Prayagraj, India; b Plant Molecular Biology, International Centre for Genetic Engineering and Biotechnology, New Delhi, India

**Keywords:** G protein-coupled receptor, GTPase, heterotrimeric G proteins, interactome mapping, membrane yeast-two-hybrid

## Abstract

G protein-coupled receptors (GPCRs) constitute the largest family of transmembrane proteins in metazoans that mediate the regulation of various physiological responses to discrete ligands through heterotrimeric G protein subunits. The existence of GPCRs in plant is contentious, but their comparable crucial role in various signaling pathways necessitates the identification of novel remote GPCR-like proteins that essentially interact with the plant G protein α subunit and facilitate the transduction of various stimuli. In this study, we identified three putative GPCR-like proteins (OsGPCRLPs) (LOC_Os06g09930.1, LOC_Os04g36630.1, and LOC_Os01g54784.1) in the rice proteome using a stringent bioinformatics workflow. The identified OsGPCRLPs exhibited a canonical GPCR ‘type I’ 7TM topology, patterns, and biologically significant sites for membrane anchorage and desensitization. Cluster-based interactome mapping revealed that the identified proteins interact with the G protein α subunit which is a characteristic feature of GPCRs. Computational results showing the interaction of identified GPCR-like proteins with G protein α subunit and its further validation by the membrane yeast-two-hybrid assay strongly suggest the presence of GPCR-like 7TM proteins in the rice proteome. The absence of a regulator of G protein signaling (RGS) box in the C- terminal domain, and the presence of signature motifs of canonical GPCR in the identified OsGPCRLPs strongly suggest that the rice proteome contains GPCR-like proteins that might be involved in signal transduction.

## 1. Introduction

The ability to sense the environment and respond appropriately to it is crucial for the survival of the organisms. G protein-coupled receptors (GPCRs) are the largest, ubiquitous and most pliable heptahelical transmembrane ‘receptor’ proteins that transduce diverse forms of extracellular stimuli ranging from ions, light, temperature, odorants, proteins, nucleotides, or neurotransmitters into the intracellular environment in conjugation with an intracellular heterotrimeric G protein ‘transducer’ complex (Gαβγ) subsequently activating the majority (80%) of downstream signaling cascades to maintain cellular homeostasis.^1–5^ The classical paradigm of GPCR-mediated signaling initiates with the binding of ligands to their cognate GPCRs and induces conformational transformations in the receptor. Ligand-bound transitional GPCRs function as guanine nucleotide exchange factors (GEFs) for the GDP-bound Gα subunit promoting the exchange of GDP for GTP and culminating in the dissociation of activated Gα (Gα-GTP) and free Gβγ dimers.^6,7^ Activated Gα-GTP and free Gβγ dimers independently induce diverse downstream effector protein cascades including enzymes (adenylyl cyclases, Protein kinases etc.), ion selective channels (Ca^+^-K^+^ channel, Na^+^-H^+^ exchange), effector enzymes (phospholipase C and D) and secondary messengers (diacyl glycerol and inositol 1,4,5-trisphosphate).^4,8-11^ Post signal relay, deactivation of the G protein signaling pathway is mediated by the intrinsic GTPase activity of Gα-GTP to reconstitute the Gαβγ heterotrimer. The rate of Gα-GTP hydrolysis is G protein subfamily-specific. The heptahelical transmembrane regulators of G protein signaling (RGS) proteins function as GTPase-accelerating proteins (GAPs), negative regulators of G protein signaling, for specific Gαs and regulate the magnitude and endurance of signaling responses initiated by GPCRs.^7,12–16^

Members of the GPCR protein family share a unique core domain composed of 7–9 transmembrane (TM) helices connected through three extracellular loops (ECLs) and three intracellular (ICLs) with an extracellular N-terminus with ligand-binding domain, and a C-terminus cytosolic tail, a distinct characteristic not observed in other classes of cell membrane receptors.^17–19^ The heterotrimeric G protein-mediated signal transduction pathway is transversely conserved in metazoans, and readily apparent in plants.

The human proteome is reported to harbor >850 GPCRs possessing homo/heterodimerization properties and has a rich repertoire of G proteins comprising 23 Gα, 5 Gβ, and 12 Gγ subunits^20–22^ leading to over 1,300 theoretical heterotrimeric complexes. This large repertoire creates extensive signaling pathways to fulfill the response to diverse environmental stimuli. In sharp contrast, the number of known heterotrimeric signaling complex components in plants is dramatically lower. *A. thaliana* has only 1 canonical Gα, 1 Gβ, two canonical Gγ, one unique RGS, and one canonical orphan GPCR.^23–25^ In plants, G protein functions are well-defined in fungal defense,^26–28^ seed germination,^29,30^ stomatal opening and closing^31–33^ oxidative stress,^34,35^ and sugar precipitation.^29,30,36^ However, no receptor for signal transduction from the environment to the cell has been reported. Whole-genome sequencing revealed that heterotrimeric G protein signaling and its complexes are highly complex. A single putative GPCR (GCR1) was identified and experimentally investigated in Arabidopsis.^37–40^ Studies of the few plant GPCRs characterized to date have provided evidence that plants use similar signaling components to regulate G protein-mediated signaling, although the signal inputs are different.^9^^–41–44^

The significantly reduced sequence homology (<25%) among GPCRs within a single GPCR family as well as across distinct GPCR families, impedes the identification of GPCRs in other life forms.^45^ Hence, the identification of novel GPCRs based on their two-dimensional topology, which classically consists of an extracellular amino terminus, seven membrane-spanning domains connected by three ECLs, and three ICLs and an intracellularly located C-terminal tail possessing the ability to couple with the Gα subunit of the heterotrimeric G protein, seems more appropriate. Recent advancements in whole-genome sequencing and comprehensive bioinformatics approaches have provided an alternative to expedite the mining of large genomes or proteomes for novel GPCRs.

The two most promising software programs for proteome-wide mining of seven helical transmembrane proteins are: the quasi-periodic feature classifier (QFC) algorithm,^46^ which is used for the identification of GPCRs and other multi-transmembrane proteins from genome databases using statistically characterized local and global structural feature-based ‘feature space’ to determine the quasi-periodic physicochemical properties of multi-transmembrane proteins. 7TMRmine^47^ is the other powerful tool for filtering the whole proteome via alignment-free and alignment-based classifiers. It also helps to study the diversity and phylogenetic relationships between GPCRs. The signature functional features of GPCRs have been established by earlier studies. The topology of a protein is an important feature, and various bioinformatics platforms can be used to predict the topology of any membrane protein. HMMTOP,^48^ Phobius,^49^ and TMpred^50^ are significant tools used for topology prediction. Improved transmembrane protein topology prediction tools with deep transfer learning reduce the dependence on evolutionary information.^51^

In the present study, we utilized pre-established experimental information and respective databases for the screening of putative GPCRs from the rice proteome. The experimental workflow was designed to address all known functional characteristics of GPCRs, starting from proteome-wide screening followed by topological analysis, functional analysis, biologically significant site prediction, motif scanning, and protein network analysis, to understand the fate of the proteins. The identified high-ranked GPCR-like proteins were tested for their interaction with the rice Gα subunit. Our study provides strong evidence for the presence of at least three 7TM noncanonical OsGPCRLPs in rice proteome that interact with rice Gα heterotrimeric subunit.

## 2. Materials and methods

### 2.1. Computational search for G protein-interacting 7TM receptor(s)

#### 2.1.1. Proteome-wide computational mining of 7TM receptor(s)-like proteins

**Figure 1.**
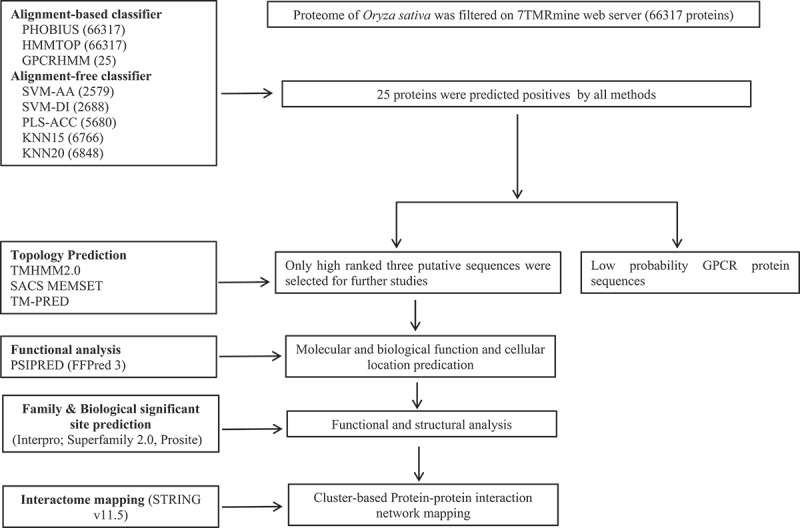
Flowchart detailing the computational analysis of candidate GPCRLPs in rice.

The proteome of *O. sativa* subsp. *indica* was scanned for hierarchical identification of heptahelical transmembrane receptor (7TMR) proteins on the 7TMRmine integrated web server (http://bioinfolab.unl.edu/emlab/7tmr/),^47^ which provides flexibility to integrate alignment-based and alignment-free protein classifiers in any combination and hierarchical order (Figure 1). The alignment-based protein classification method relies on a homology search of query 7TMRs with well-characterized protein families and is less effective at identifying unrelated or novel 7TMRs. However, alignment-free classifiers are based on multivariate statistical methods that identify remote similarities and divergent 7TMRs with greater sensitivity.^47^ We used a combinatorial approach comprising alignment-based (Phobius, HMMTOP, and GPCRHMM) and alignment-free (SVM-AA, SVM-DI, PLS-ACC, KNN15, KNN20) classifiers to predict the most potential 7TMR candidates in the rice proteome. All the ‘positive’ filtered loci at each level were combined through the ‘AND’ approach and further subjected to topology analyses.

#### 2.1.2. Topology prediction of high-ranked putative OsGPCRLPs

The positive 7TM protein sequences obtained by the combinatorial approach on 7TMRmine were further analyzed for their 2D topology and number of transmembrane domains on TMHMM2.0 (https://services.healthtech.dtu.dk/service.php?TMHMM-2.0.), SACSMEMSET (http://www.sacs.ucsf.edu/cgi-bin/memsat.py.) and TOPCONS (https://topcons.cbr.su.se/pred/). All the programs were run with the default settings.

#### 2.1.3. Functional annotation of high-ranked putative OsGPCRLPs

The PSIPRED (http://bioinf.cs.ucl.ac.uk/psipred/) workbench was used for functional annotation of the high-rank candidate OsGPCRLPs on FFPRED3, a platform for the prediction of gene ontology annotations.^52^ The amino acid sequences were used as the input data for FFPRED3. The output data included biological and molecular function and cellular component predictions.

#### 2.1.4. Superfamily, family, and biologically significant site prediction in high-ranked OsGPCRLPs

The protein superfamily and family of high-ranked putative OsGPCRLPs were determined via Interpro (https://www.ebi.ac.uk/interpro/) and SUPERFAMILY 2.0 (http://supfam.org). InterPro provides functional analysis of proteins by classifying them into families and predicting domains and important sites using predictive models available on the InterPro consortium.^53^ SUPERFAMILY is an HMM-based database for structural and functional annotation of proteins and genomes.^54^ Biologically significant sites were analyzed on PROSITE (https://prosite.expasy.org/), a protein domain and family database to identify significant sites, patterns, and profiles.^55,56^

#### 2.1.5. Interactome mapping of high-ranked candidate OsGPCRLPs

The potential interacting partners of all high-ranked putative OsGPCRLPs were mapped on STRING v.11.5^57^ with the *O. sativa* dataset. STRING is a database for the prediction of protein interaction networks based on established and predicted physical (direct) as well as functional (indirect) protein-protein interactions derived mainly from five data sources: genomic context predictions, high-throughput laboratory experiments, conserved co-expression, automated text-mining, and interactions aggregated from other primary databases. Interacting partners were mapped at a medium confidence score of 0.4, and there were 10 and 30, maximum interactions in 1^st^ and 2^nd^ shells, respectively. The interaction network obtained for the query proteins was then subjected to *k-means* clustering by selecting ‘3’ clusters.

#### 2.1.6. Coupling specificity prediction for high-ranked OsGPCRLPs

The coupling specificity of high-ranked putative OsGPCRLPs with four families of Gα proteins was predicted using the PRED-COUPLE 2.0 program (http://athina.biol.uoa.gr/bioinformatics/PRED-COUPLE2/).^58,59^ A safe cutoff of 0.3 was applied to discriminate between positive and negative predictions. Only loci with cutoff values above 0.3 were considered to have positive predictions.

#### 2.1.7. GPCR-like proteins prediction in high-ranked OsGPCRLPs

Prediction of GPCR-like proteins for the high-ranked OsGPCRLPs was done using BLAST search performed for ‘GPCRs only’ on gpDB (http://bioinformatics.biol.uoa.gr/gpDB), a relational repository of known GPCRs, G proteins, and effectors classified into respective classes, families and subfamilies.^60^ The BLAST output lists the sequences in the database having significant E-values ranked by statistical significance.

#### 2.1.8. Prediction of ‘RGS box’ architecture in high-ranked OsGPCRLPs

Multiple sequence alignment of the cytoplasmic C-terminal domain of high-ranked OsGPCRLPs was performed with human RGS19 (hRGS19; SwissProt accession no. P49795), Arabidopsis RGS1 (AtRGS1; GenPept NP_189238) and known plant GPCRs from Arabidopsis (AtGCR1; accession no. AAN15633.1,^38^ lotus (LjGCR1; UniProtKB/TrEMBL B6GV49,^61^ and pea (PsGPCR; NCBI protein id AAY30370.2;^43^ on Clustal Omega (https://www.ebi.ac.uk/Tools/msa/clustalo) under default settings.

This alignment was performed to evaluate the presence of RGS boxes in high-ranked OsGPCRLPs and/or their close relatedness with known plant GPCRs. The C-terminal domains of high-ranked OsGPCRLPs and other plant GPCRs were generated by TMHMM 2.0, while the sequences used for AtRGS1 and hRGS19 were chosen according to Chen et al.^36^

### 2.2. In vivo coupling validation of computational protein-protein predictions in high-ranked OsGPCRLPs

High-ranked candidate OsGPCRLPs were further validated through a membrane-based split-ubiquitin yeast-2-hybrid (MYTH) system^62^ to establish their protein-protein interaction with the rice heterotrimeric G protein α subunit (RGA1). MYTH is a split-ubiquitin-based Y2H system specialized for membrane proteins that exploit the inherent ability of the two termini of the wild-type split ubiquitin molecule (NubI and Cub) to undergo spontaneous assembly followed by subsequent Ubiquitin binding protein (UBP)-catalyzed cleavage of transcription factor fused downstream of Cub. A single amino acid substitution of Ile with Gly at position 13 attenuates this spontaneity, which is then dictated by the interactions between the ‘bait’ and ‘prey’ proteins fused with Cub and Nub, respectively. Interactions between the bait and prey proteins facilitate the reconstitution of the split-ubiquitin heterodimer, which upon recognition by UBP, is cleaved at its C-terminus to release the transcription factor LexA/VP16. This cleaved transcription factor then translocates into the nucleus, where the DNA-binding domain of LexA binds to its operator upstream of reporter genes (in this case, *His*, *Ade2*, and *lacZ*), promoting VP16-mediated transcriptional activation of reporter genes.

#### 2.2.1. Strains, media

All constructs were expressed in the *Saccharomyces cerevisiae* strain NMY51 (*MATahis3∆200 trp1–901 leu2–3, 112 ade2 LYS2*: (lexAop)_4_ -*HIS3 ura3*: (lexAop)_8_-lacZ (lexAop)_8_-ADE2 GAL4). Yeast cells were subsequently grown in YAPD media following standard microbial procedures. Transformed yeast cells were selected and screened through auxotrophic selection on defined minimal media of successively increasing stringency from double dropout (2D) (YNB-trp-leu) transformation selection medium (TSM) to triple dropout (3D) (YNB-trp-leu-his), quadruple dropout (4D) (YNB-trp-leu-his-ade) and quadruple dropout supplemented with 0.5 mM 3-amino-1,2,4-triazole (3-AT) interaction selection media (ISM).

#### 2.2.2. Construction of plasmids

**Figure 2.**
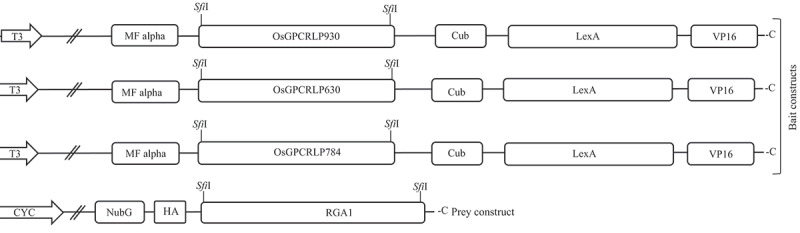
Schematic line diagram representing the construct design for MYTH assay.

All the bait proteins (OsGPCRLP930, OsGPCRLP784, and OsGPCRLP630) were cloned and inserted into pTMBV-α (bait vector) at the two ‘*Sfi*I’ restriction sites, ensuring in-frame insertion with the downstream ‘Cub-LexA/VP16’ tag to encode the C-terminally tagged bait ‘OsGPCRLP-Cub-LexA/VP16’. The stop codons of all bait genes were deleted to ensure the synthesis of the Cub-LexA/VP16 tag. Similarly, the prey protein RGA1 was cloned and inserted into pPR3Nα (prey vector) at the *Sfi*I restriction sites such that the fusion protein NubG-RGA1, which is encoded by the prey vector, contains NubG at its N-terminus (Figure 2). The control prey vectors pOstNubI and pOstNubG, which encode a fusion of yeast ER-localized membrane protein oligosaccharyl transferase to wild-type and modified ‘Nub’ yeast ubiquitin, respectively, were used as positive and negative controls.^63^ Cloning was performed using standard cloning procedures, and all the clones were confirmed through restriction profiling and sequencing. The sequences of primers used for cloning bait and prey proteins are listed in Table 1.

**Table 1.** Primers used for cloning of OsGPCRLPs in MYTH vectors.

Name	Sequence
Primers for Bait vector construction	
TMBV930 For	5′-AGGCCATTACGGCCCAATGGCGGCATCGGCGGCG-3′
TMBV930 Rev	5′-TGGCCGAGGCGGCCGGTGTGTTACTCGCATCGACAATAA-3′
TMBV630 For	5′-GGCCATTACGGCCCAATGCACCCGATAACATTTTTCAC-3′
TMBV630 Rev	5′-GGCCGAGGCGGCCGGCAGGGCAATGGAATGACTTGTT-3′
TMBV784 For	5′-AGGCCATTACGGCCCAATGGGCCGCCACGGCATGT-3′
TMBV784 Rev	5′-TGGCCGAGGCGGCCGGAATAGGGCTTGACTTGAGGGC-3′
Primers for prey vector construction	
pPR3Nα RGA1 For	5′-AGGCCATTACGGCCATGTCCGTGCTTACCTGTGT-3′
pPR3Nα RGA1 Rev	5′-AGGCCGAGGCGGCCTCAAGTTCCTTCCCTGGAGC-3′

#### 2.2.3. Expression of the plasmid constructs

The functional expression of the OsGPCRLPs and bait-prey interactions were established in the reporter strain NMY51 as described by Iyer et al.^64^ with modifications. Yeast transformation was performed following the LiOAc/ssDNA/PEG protocol.^65^ The bait and prey vector combinations (~1.5 µg each) used for cotransformation are listed in Table 2. Before total protein extraction, the yeast cells were treated with LiAc and NaOH.^66^ The isolated total protein was resolved through SDS-PAGE (10% resolving and 5% stacking), and the gel was stained with 0.25% Coomassie Brilliant Blue *R*-250. Furthermore, western blot analysis was performed to verify bait expression following the standard protocol. Primary anti-LexA antibody (1:1500) and alkaline phosphatase-conjugated secondary anti-rabbit IgG (1:10000) were used. The functionality of the bait was validated via bait and pOstNubI (TI) coexpression. The probability of bait self-activation was assayed through the coexpression of bait with the negative control prey vector, pOstNubG (TG). Nontransformed yeast cells were used as experimental negative controls. Transformants were selected and screened for positive interactions on auxotrophic selection plates of increasing stringency (2D→3D→4D). To test the proper activation of reporter genes, 4D selection plates were supplemented with 0.5 mM 3-AT. Furthermore, the interaction strength for each of the three bait-prey combinations was assessed using a filter liftoff assay involving the activation of the *lacZ* reporter gene and color-based detection of β-galactosidase activity.

**Table 2.** Vector combinations used in transformation of reporter strain NMY51.

	Bait	Prey	Coding
Positive controls	930TMBV	pOstNubI	930TI
	630TMBV	pOstNubI	630TI
	784TMBV	pOstNubI	784TI
Negative controls	930TMBV	pOstNubG	930TG
	630TMBV	pOstNubG	630TG
	784TMBV	pOstNubG	784TG
Experimental construct combinations	930TMBV	pPR3Nα	930TN
	630TMBV	pPR3Nα	630TN
	784TMBV	pPR3Nα	784TN

## 3. Results

### 3.1. Computational search for 7TMR(s) in rice proteome

#### 3.1.1. Proteome-wide mining identified 7TMR(s) in rice proteome

**Figure 3.**
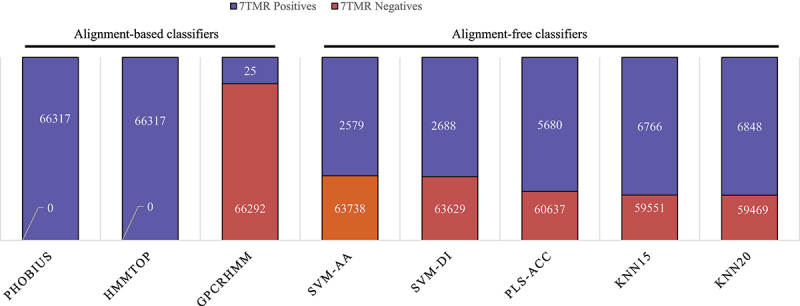
Proteome-wide mining for heptahelical transmembrane GPCRLPs on 7TMR mine.

On alignment-based classifiers Phobius and HMMTOP identified all proteins as 7TMR positives, whereas, GPCRHMM classified 25 proteins as 7TMR positives, while remaining as negatives (Figure 3). The alignment-free classifiers *viz*. SVM-AA classified 2579 as 7TMR and 63,738 as non-7TMR proteins, SVM-DI as 2688 positives and 63,629 negatives, PLS-ACC as 5680 positives and 60,637 negatives, KNN15 as 6766 positives and 59,551 negatives, and KNN20 as 6848 positives and 59,469 negative 7TMRs (Figure 3). The combinatorial approach, including both alignment methods, identified 25 7TMR positive proteins that may be GPCRLP candidates. These candidates were considered for further analyses, and their loci, length and predicted signal peptides are listed in Table 3.

**Table 3.** Proteome-wide identification of candidate GPCRLPs in rice on 7TMRmine server.

Locus	Length (aa)	Signal peptide	Phobius		HMMTOP		GPCRHMM	SVM-AA	SVM-DI	PLS-ACC	KNN15	KNN20
LOC_Os10g02350.1	585	Y	9	OUT	9	OUT	G	G	G	G	G	G
LOC_Os10g05690.1	547	N	10	OUT	12	IN	G	G	G	G	G	G
LOC_Os04g36630.1	318	N	7	OUT	7	OUT	G	G	G	G	G	G
LOC_Os04g01520.1	413	N	9	IN	9	IN	G	G	G	G	G	G
LOC_Os01g66190.1	397	N	7	OUT	7	OUT	G	G	G	G	G	G
LOC_Os01g66190.2	397	N	7	OUT	7	OUT	G	G	G	G	G	G
LOC_Os01g61970.1	449	Y	7	OUT	9	OUT	G	G	G	G	G	G
LOC_Os01g54784.1	361	N	7	OUT	7	OUT	G	G	G	G	G	G
LOC_Os03g13380.1	595	Y	9	OUT	10	IN	G	G	G	G	G	G
LOC_Os02g54990.2	414	Y	9	OUT	10	IN	G	G	G	G	G	G
LOC_Os02g01100.1	574	N	11	IN	11	IN	G	G	G	G	G	G
LOC_Os02g01100.2	571	N	11	IN	11	IN	G	G	G	G	G	G
LOC_Os02g01100.3	571	N	11	IN	11	IN	G	G	G	G	G	G
LOC_Os02g02750.3	325	N	7	OUT	6	IN	G	G	G	G	G	G
LOC_Os02g34690.1	590	Y	9	OUT	10	IN	G	G	G	G	G	G
LOC_Os09g38530.1	646	Y	9	OUT	10	IN	G	G	G	G	G	G
LOC_Os06g09930.1	321	Y	7	OUT	7	OUT	G	G	G	G	G	G
LOC_Os06g04130.1	454	Y	7	OUT	9	OUT	G	G	G	G	G	G
LOC_Os06g04130.2	454	Y	7	OUT	9	OUT	G	G	G	G	G	G
LOC_Os06g44140.1	645	Y	9	OUT	10	IN	G	G	G	G	G	G
LOC_Os12g38570.1	594	N	11	IN	11	IN	G	G	G	G	G	G
LOC_Os12g07670.1	598	Y	9	OUT	9	OUT	G	G	G	G	G	G
LOC_Os05g39730.1	554	N	7	OUT	7	OUT	G	G	G	G	G	G
LOC_Os05g38720.1	458	Y	7	OUT	8	IN	G	G	G	G	G	G
LOC_Os11g07910.1	593	Y	9	OUT	10	IN	G	G	G	G	G	G

#### 3.1.2. Topology prediction identified canonical GPCR-type topology

Topology prediction identified three proteins LOC_Os06g09930.1 (OsGPCRLP930), LOC_Os01g54784.1 (OsGPCRLP784), and LOC_Os04g36630.1 (OsGPCRLP630), exhibiting typical GPCR-type ‘type-I’ heptahelical transmembrane topology on all three topology prediction tools under study and were classified as high-ranking candidate GPCRLPs. Similarly, 7 loci were categorized as medium-ranked candidate GPCRLPs that met the above criteria according to at least one topology prediction tool. LOC_Os01g66190.1, LOC_Os01g66190.2, and LOC_Os05g38720.1 showed typical GPCR topology on two tools, while LOC_Os06g04130.1, LOC_Os06g04130.2, LOC_Os01g61970.1 and LOC_Os02g02750.3 were positive for only TOPCONS. The remaining 15 locus-encoded proteins were classified as low-ranking candidate GPCRLPs (Table 4). Hereafter, further studies were performed only with high-ranked candidate GPCRLPs which were annotated as OsGPCRLP930, OsGPCRLP784, and OsGPCRLP630.

**Table 4.** Topology prediction.

	Locus	Length (aa)	TMHMM 2.0		SACS MEMSET		TOPCONS		
			N-Ter Topology	TM	N-Ter Topology	TM		N-Ter Topology	TM
High rank									
1	LOC_Os06g09930.1	321	OUT	7	OUT	7	OUT		7
2	LOC_Os04g36630.1	318	OUT	7	OUT	7	OUT		7
3	LOC_Os01g54784.1	361	OUT	7	OUT	7	OUT		7
Medium rank									
4	LOC_Os01g66190.1	397	OUT	7	IN	8	OUT		7
5	LOC_Os01g66190.2	397	OUT	7	IN	8	OUT		7
6	LOC_Os05g38720.1	458	OUT	7	OUT	7	OUT		6
7	LOC_Os06g04130.1	454	IN	7	OUT	8	OUT		7
8	LOC_Os06g04130.2	454	IN	7	OUT	8	OUT		7
9	LOC_Os01g61970.1	449	IN	8	OUT	6	OUT		7
10	LOC_Os02g02750.3	325	OUT	6	IN	8	OUT		7
Low rank									
11	LOC_OS10g02350.1	585	IN	9	IN	10	OUT		9
12	LOC_Os10g05690.1	547	OUT	10	OUT	12	OUT		10
13	LOC_Os04g01520.1	413	IN	9	IN	9	IN		9
14	LOC_Os03g13380.1	595	OUT	10	IN	9	OUT		9
15	LOC_Os02g54990.2	414	IN	10	IN	10	IN		10
16	LOC_Os02g01100.1	574	OUT	10	OUT	1	IN		11
17	LOC_Os02g01100.2	571	OUT	10	IN	1	IN		9
18	LOC_Os02g01100.3	571	OUT	10	IN	1	IN		9
19	LOC_Os05g39730.1	554	IN	6	OUT	6	IN		6
20	LOC_Os02g34690.1	590	OUT	10	OUT	9	OUT		9
21	LOC_Os09g38530.1	646	IN	10	IN	10	OUT		11
22	LOC_Os06g44140.1	645	IN	10	IN	10	OUT		11
23	LOC_Os12g38570.1	594	OUT	10	OUT	11	IN		9
24	LOC_Os12g07670.1	598	OUT	9	OUT	9	OUT		9
25	LOC_Os11g07910.1	593	IN	10	IN	4	OUT		9

OUT = N-terminus is extracellularly located; IN = N-terminus is intracellularly located; Shaded loci possess typical GPCR-type ‘type-I’ heptahelical transmembrane topology.

#### 3.1.3. Functional annotation of high-rank OsGPCRLPs

All high-ranked OsGPCRLPs were shown to be involved in cell communication and signal transduction in response to various stimuli and are associated with cell surface receptor signaling pathways. Notably, the entire candidate OsGPCRLPs were identified as having biological and molecular functions in GPCR signaling pathways. However, the OsGPCRLP630 did not show GPCR activity unlike the other two candidates. All the candidate GPCRLPs were identified as intrinsic components of the plasma membrane. Unlike OsGPCRLP930 and OsGPCRLP630, the candidate OsGPCRLP784 was not reported to be a component of the nuclear outer membrane-ER membrane network. All the candidates exhibited significant probabilities and high (H) SVM reliability for the biological, molecular, and cellular component features (Table 5).

**Table 5.** Functional annotation of high-ranked OsGPCRLPs using FFPRED3.

Name	GO term	Probability/SVM Reliability		
		OsGPCRLP930	OsGPCRLP784	OsGPCRLP630
**Biological function**				
Response to stimulus	GO:0050896	0.937/L	0.937/L	0.946/L
G protein-coupled receptor signaling pathway	GO:0007186	0.881/H	0.913/H	0.516/H
Cell surface receptor signaling pathway	GO:0007166	0.860/H	0.890/H	0.766/H
Signal transduction	GO:0007165	0.852/L	0.893/L	0.812/L
Cellular response to stimulus	GO:0051716	0.837/L	0.836/L	0.844/L
Detection of chemical stimulus involved in sensory perception	GO:0050907	–	–	0.926/H
Cell communication	GO:0007154	0.820/L	0.831/L	0.763/L
Regulation of signal transduction	GO:0009966	0.605/L	0.605/L	0.588/L
**Molecular function**				
Receptor activity	GO:0004872	0.949/H	0.948/H	0.870/H
Signaling receptor activity	GO:0038023	0.908/H	0.911 H	0.800/H
Transmembrane signaling receptor activity	GO:0004888	0.888/H	0.948/H	0.664/H
G protein-coupled receptor activity	GO:0004930	0.878/H	0.888/H	–
Signal transducer activity	GO:0004871	0.804/H	0.792/H	0.754/H
G protein-coupled receptor binding	GO:0001664	0.591/H	0.722/H	–
**Cellular Component Predictions**				
Intrinsic component of membrane	GO:0031224	1.000/H	1.000/H	1.000/H
Membrane	GO:0016020	0.968/H	0.971/H	0.967/H
Plasma membrane	GO:0005886	0.909/H	0.926/H	0.918/H
Integral component of plasma membrane	GO:0005887	0.817/H	0.857/H	0.935/H
Intrinsic component of plasma membrane	GO:0031226	0.768/H	0.818/H	0.772/H
Endomembrane system	GO:0012505	0.754/H	0.758/H	0.754/H
Nuclear outer membrane-endoplasmic reticulum membrane network	GO:0042175	0.560/H	–	0.667/H
Organelle membrane	GO:0031090	–	–	0.801/H

H = high, L = low SVM reliability for biological, molecular and cellular components' feature.

#### 3.1.4. Candidate OsGPCRLPs belonged to GPCR family possessing biologically significant sites

InterPro identified OsGPCRLP930 as a member of the GCR1-cAMP receptor family (IPR022343: InterPro family) with GCR1-cAMP receptor (PR02001: PRINTS) conserved motifs (32–50, 51–66, 82–100, 223–241 and 255–274 aa) and GCR1 family (IPR022340: InterPro family) with putative plant GPCR and GCR1 (PR02000: PRINTS) signature motifs (21–36, 38–52, 54–76, 84–97, 99–117, 122–137, 161–173, 174–197, 217–238, 247–264 and 265–281 aa). The OsGPCRLP930 protein sequence from amino acids 21–275 represents domains corresponding to GPCR_2-like_TM (IPR017981: InterPro domains) and G protein-coupled receptor family 2 profile 2 (PS50261: PROSITE profiles). Similarly, the OsGPCRLP784 was categorized as a member of the THH1/TOM1/TOM3 family (IPR040226) with TOBAMOVIRUS MULTIPLICATION PROTEIN 1-LIKE ISOFORM X1 (PTHR31142, PANTHER) signature motif from amino acids 26–340. Additionally, amino acids 28–135 and 182–321 of putative OsGPCRLP784 represent the THH1/TOM1/TOM3 protein domain (InterPro: IPR009457 and Pfam: PF06454). However, the gene product of OsGPCRLP630 belonged to uncharacterized conserved protein UCP031277 (IPR016971) family with the UCP031277 (PIRSF031277; PIRSF) signature motif from amino acids 1–317. SUPERFAMILY 2.0 classified OsGPCRLP930 and OsGPCRLP784 under the Family A GPCR-like superfamily and Rhodopsin-like family with significant E-values, whereas no result was obtained for OsGPCRLP630 (Table 6).

**Table 6.** Prediction of superfamily and family of candidate OsGPCRLPs.

Gene ID	InterPro		SUPERFAMILY 2.0			
	Family (InterProID)	Domain (aa)	Superfamily (aa)	E-value	Family	E-value
OsGPCRLP930	GCR1-cAMP receptor (IPR022343); G protein-coupled receptor GCR1 putative (IPR022340)	GPCR_2-like_TM, IPR017981 G protein-coupled receptors family 2 PS50261 (21–275)	Family A GPCR-like (29–286)	3.3 × 10^15^	Rhodopsin-like	0.023
OsGPCRLP784	THH1/TOM1/TOM3; (IPR040226)	THH1/TOM1/TOM3_ dom IPR009457, PF06454 (28–135 and 182–321)	Family A GPCR -like (168–309)	0.011	Rhodopsin-like	0.02
OsGPCRLP630	Uncharacterized conserved protein UCP031277 (IPR016971)	UCP031277 IPR016971, PIRSF031277 (1–317)	No results			

**Figure 4.**
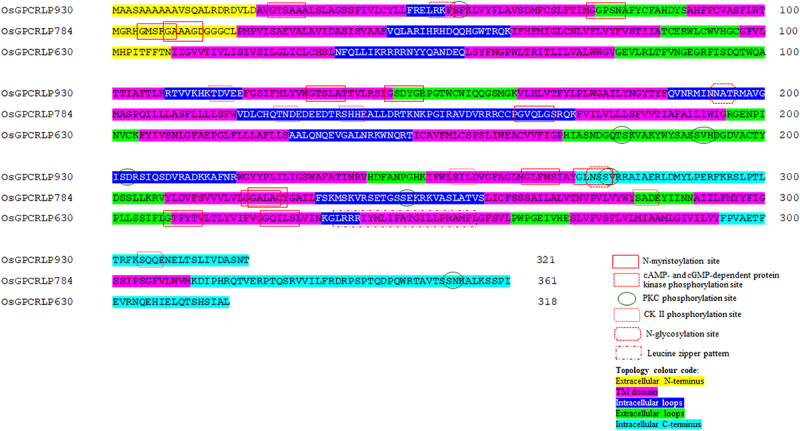
Biologically significant sites in high-ranked OsGPCRLPs analyzed on PROSITE.

The ExPASy PROSITE database of protein families and domains identified various patterns and biologically significant sites in the OsGPCRLPs (Figure 4). In OsGPCRLP930, six N-myristoylation sites (25–30: GTsaAA; 74–79: GGpsNA; 131–136: GTslAT; 143–148: GSdyGR; 264–269: GLfnSI; 272–277: GLnsSV), one cAMP- and cGMP-dependent protein kinase phosphorylation site (50–53: RKfS), three protein kinase C (PKC) phosphorylation sites (53–55: SfK; 202–204: SdR; 276–278: SvR), three casein kinase (CK) II phosphorylation sites (116–119: TdvE; 253–256: SilD; 305–308: SqqE), and two N-glycosylation sites (193–196: NATR; 274–277: NSSV) were identified. In OsGPCRLP784, five N-myristoylation sites (5–10: GMsrGA; 9–14: GAaaGD; 163–168: GVqlGS; 221–226: GGalAC; 222–227: GAlaCY), three casein kinase (CK) II phosphorylation sites (126–129: TndE; 136–139: ShhE; 281–284: SadE), and two PKC phosphorylation sites (245–247: SeK; 352–354: SnK) were identified. The OsGPCRLP630 contained one PKC phosphorylation site (178–180: TsK), one casein kinase (CK) II phosphorylation site (190–193: SvhD), two N-myristoylation sites (209–214: GTfyTV; 224–229: GQilSL), and one leucine zipper pattern (235–256: LrrriymLifatgiLlpramfL).

#### 3.1.5. Interactome mapping of high-ranked candidate OsGPCRLPs revealed interactions with G protein-mediated signaling network partners

STRING analyses mapped 41, 31, and 34 interacting proteins for the OsGPCRLP930, OsGPCRLP784, and OsGPCRLP630 query proteins, respectively (Supplementary Tables S1–3). Proteins that are directly linked to GPCR functions are shown in Figure 5. Interactome mapping generated a large number of interacting partners for each of the query proteins clustered in three distinct clusters, with colors indicating no functional priority mentioned in the supplementary tables . Proteins sharing similar types of interactions with the query protein are usually grouped into a single cluster. For OsGPCRLP930, 16 proteins were grouped in red and green, whereas 9 proteins were grouped in the blue cluster (Supplementary Table S1). The OsGPCRLP930 interacted with the G protein components viz. RGA1, RGB1, RGG1, RGG2, and the putative XLG of the red cluster (Figure 5(a)). However, the OsGPCRLP784 exhibited interactions with 9, 18, and 4 proteins grouped into red, green, and blue clusters, respectively (Supplementary Table S2). OsGPCRLP784 also interacted with G protein components similar to OsGPCRLP930, but not with XLG (Figure 5(b)). The interaction network of the OsGPCRLP630 contained 31 proteins in red, 1 protein in green, and 2 proteins in the blue cluster (Supplementary Table S3). The OsGPCRLP630 also interacted with all the G protein subunits, except RGG2 and XLG, as mapped in the red cluster (Figure 5(c)). In addition to interacting with G protein subunit components, OsGPCRLP630 and OsGPCRLP784 interact with the GPCR-type G protein (GTG) COLD1. In addition, two uncharacterized protein kinase-domain-containing proteins (B8B954 and B8BDL3) were also mapped to OsGPCRLP630.

**Figure 5a.**
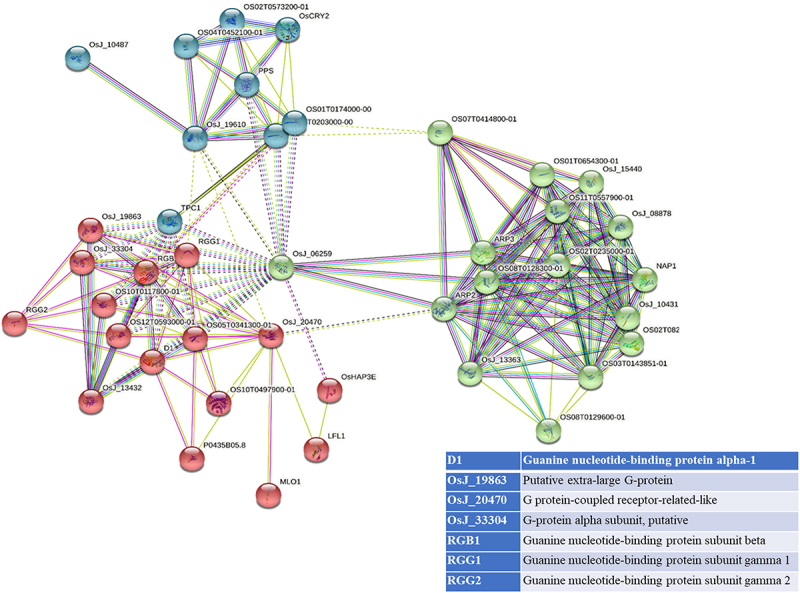
Interactome mapping for high-ranked OsGPCRLPs on String v.11.5. 5a representing the interactome map of OsGPCRLP930 for 40 proteins. 5b representing the interactome map for OsGPCRLP784 for 30 proteins. 5c representing the interactome map for OsGPCRLP630 for 33 proteins. Each map includes one query protein. The K-means clustering function is distributed for all proteins in three clusters (Red, Green, Blue) (Supplementary data).

**Figure 5b.**
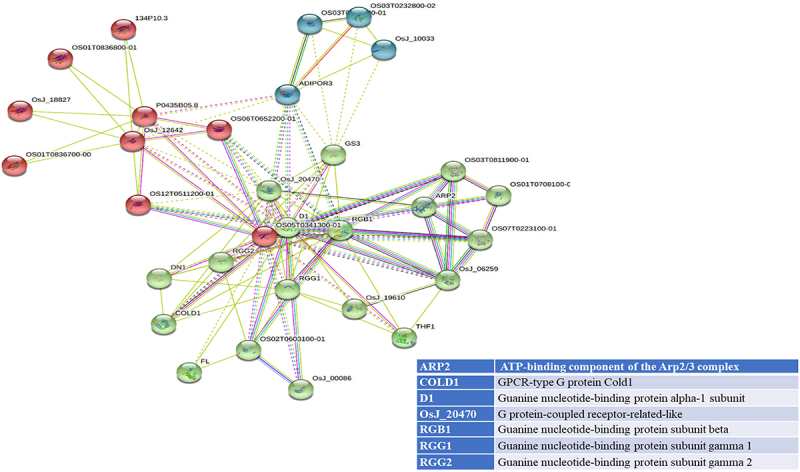
Continued.

**Figure 5c.**
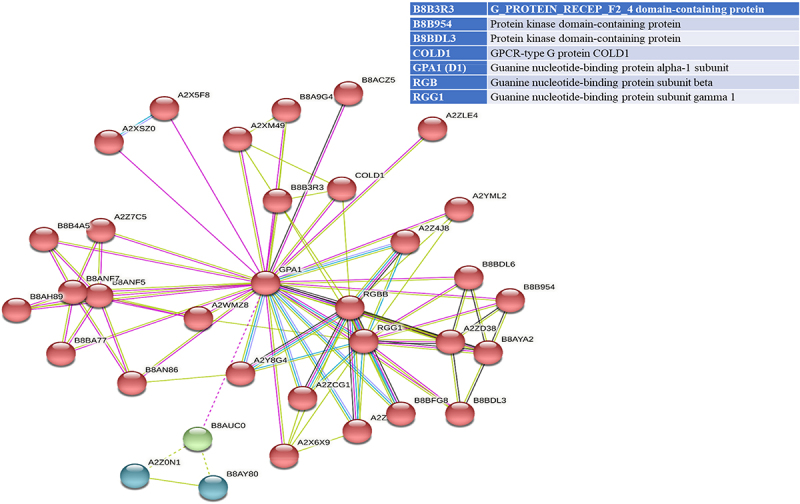
Continued.

#### 3.1.6. Coupling specificity predicted Gα class-specific interactions

The normalized posterior probability scores of OsGPCRLP930 were 0.93, 0.67 and 0.38 for *Gα*_*i/o*_, *Gα*_*q/11*_ and *Gα*_*s*_, respectively. Similarly, the coupling specificity scores of OsGPCRLP630 were 0.78, 0.98, and 0.67 for *Gα*_*i/o*_, *Gα*_*q/11*_, and *Gα*_*s*_, respectively. On the other hand, OsGPCRLP784 showed significant coupling scores of 0.97 and 0.78 for *Gα*_*i/o*_ and *Gα*_*q/11*_, respectively. All three candidate OsGPCRLPs were predicted to have negative coupling scores for *Gα*_*12/13*_, while OsGPCRLP784 additionally had a negative score for *Gα*_*s*_ (0.1) (Table 7).

**Table 7.** G protein coupling specificity predication on PRED-COUPLE 2.0 tool.

	Normalized posterior probability score			
Gene ID	*G*_*i/o*_	*G*_*q/11*_	*G*_*s*_	*G*_*12/13*_
OsGPCRLP930	**0.93**	**0.67**	**0.38**	0.02
OsGPCRLP784	**0.97**	**0.78**	0.1	0
OsGPCRLP630	**0.78**	**0.98**	**0.67**	0.30

**Score**: ≤ 0.3 = negative, **>0.3 = positive**.

#### 3.1.7. High-ranked OsGPCRLPs showed similarity with canonical GPCRs

A BLAST search on gpDB identified GPCR-like proteins for each high-ranked OsGPCRLP with significant E-values. Only the top five hits for each OsGPCRLP are shown in Table 8. The top five GPCRLP identified for OsGPCRLP930 included cAMP receptors (1, 2, 3 and 4) of the class E cyclic nucleotide receptor family of *Dictyostelium discoideum* and the class F smoothened receptor family of *Drosophila melanogaster*-1. The top five sequences similar to OsGPCRLP784 revealed by BLAST search included Class C taste receptor putative taste receptor type 2 member 38 (T2R38) of *Mus musculus*, putative odorant receptor 59c of *Drosophila melanogaster* belonging to the largest subfamily OR of class A GPCRs, opioid and opioid-like receptor_MU opioid peptide (MOR) of *Sus scrofa* and *Bos taurus* of class A and cholecystokinin and gastrin receptor_cck2 of *Bos taurus* of class A. OsGPCRLP630 was aligned with putative gustatory receptor 10a of *D. melanogaster*, class A trace amine receptor_TaR-9 of *Rattus norvegicus*, and human olfactory receptors (6V1, 52E6, and 4A4 of class A of odorant receptor family in pairwise alignment on gpDB.

**Table 8.** BLAST output of high rank OsGPCRLPs with gpDB database. BLAST search output showing only top five GPCR-like proteins identified at gpDB with significant E-values ranked by statistical significance for each high-ranked OsGPCRLP.

Query Proteins	GPCRLPs from gpDB	Range of query alignment	Range of database sequence alignment	Number of Identities	Number of Positive Substitutions	Number of Gaps	Raw Score	Expect Value
OsGPCRLP930	gpcr2774 CYCLIC NUCLEOTIDE RECEPTOR-CYCLIC AMP RECEPTOR 4-*Dictyostelium discoideum*	39, 275	28, 262	55	108	26	152	3.20862e-11
	gpcr2773 CYCLIC NUCLEOTIDE RECEPTOR-CYCLIC AMP RECEPTOR 3-*Dictyostelium discoideum*	39, 275	41, 272	53	107	29	152	3.20862e-11
	gpcr2771 CYCLIC NUCLEOTIDE RECEPTOR-CYCLIC AMP RECEPTOR 1-*Dictyostelium discoideum*	77, 239	74, 227	42	72	25	142	4.63324e-10
	gpcr2772 CYCLIC NUCLEOTIDE RECEPTOR-CYCLIC AMP RECEPTOR 2-*Dictyostelium discoideum*	39, 275	28, 260	54	105	34	136	2.29945e-09
	gpcr2804 SMOOTHENED_smoothened_*Drosophila melanogaster*-1	90, 182	347, 440	23	36	1	82	0.00420032
OsGPCRLP784	gpcr2672 TASTE RECEPTOR_Putative taste receptor type 2 member 38 (T2R38) *Mus musculus*	231, 281	219, 274	16	29	5	61	1.33004
	gpcr1807 ODORANT RECEPTOR_Putative odorant receptor 59c_*Drosophila melanogaster*	58, 86	128, 155	11	20	1	57	3.86983
	gpcr1909 OPIOID AND OPIOID-LIKE RECEPTOR_MU OPIOID PEPTIDE (MOR)_*Sus scrofa*	230, 361	257, 400	31	62	20	57	3.86983
	gpcr1901 OPIOID AND OPIOID-LIKE RECEPTOR_MU OPIOID PEPTIDE (MOR)_*Bos Taurus*	230, 361	257, 400	31	62	20	57	3.86983
	gpcr740 CHOLECYSTOKININ AND GASTRIN RECEPTOR_CCK2_*Bos Taurus*	248, 329	83, 164	17	35	-	56	5.05415
OsGPCRLP630	gpcr924 GUSTATORY RECEPTOR_Putative gustatory receptor 10a_*Drosophila melanogaster*	211, 314	18, 126	28	45	11	57	3.28544
	gpcr2546 TRACE AMINE RECEPTOR_TaR-9_*Rattus norvegicus*-1	268, 315	209, 256	11	28	-	56	4.29092
	gpcr1666 ODORANT RECEPTOR_Olfactory receptor 6V1_Homo sapiens	254, 299	11, 51	15	28	5	56	4.29092
	gpcr1585 ODORANT RECEPTOR_Olfactory receptor 52E6_*Homo sapiens*	218, 262	134, 177	14	25	1	56	4.29092
	gpcr1514 ODORANT RECEPTOR_Olfactory receptor 4A4_*Homo sapiens*	251, 299	3, 52	16	27	1	55	5.60412

#### 3.1.8. The RGS box architecture was absent in putative OsGPCRLPs

**Figure 6.**
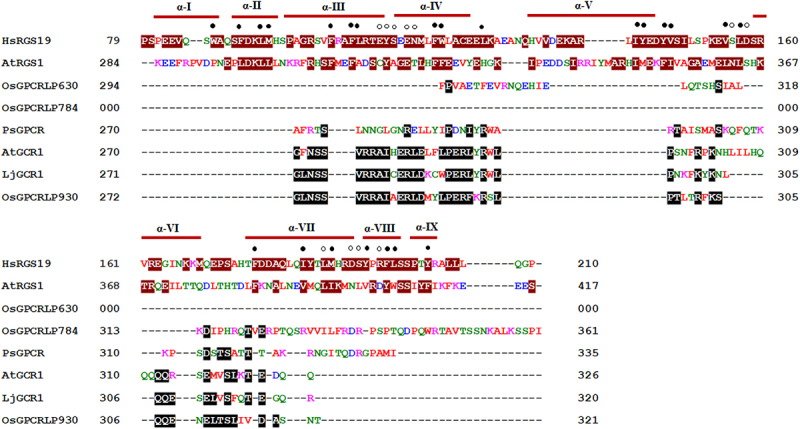
Multiple sequence alignment of cytosolic C-termini of RGS from human (HsRGS19: SwissProt accession no. P49795) and Arabidopsis (AtRGS1: GenPept NP_189238), GPCRs from pea (PsGPCR: AAY30370.2), Arabidopsis (AtGCR1: AAN15633.1), lotus (LjGCR1: UniProtKB/TrEMBL B6GV49) and high-ranked putative rice GPCRLPs (OsGPCRLP930, OsGPCRLP784 and OsGPCRLP630). Residues in white font and dark red highlight denote RGS box according to.^36^ Identical and/or similar residues in candidate OsGPCRLPs to either of known plant GPCRs are highlighted in black.

Multiple sequence alignment showed that OsGPCRLPs do not contain RGS boxes in their cytoplasmic C-terminus and do not share any similarity with the cytoplasmic C-terminal domains of Arabidopsis and human RGS proteins. However, OsGPCRLP930 showed significant sequence similarity with the Arabidopsis and lotus GCR1 proteins, while OsGPCRLP784 and OsGPCRLP630 shared few consensus residues with the plant GPCRs considered under study (Figure 6).

### 3.2. Putative OsGPCRLPs strongly coupled with RGA1

**Figure 7.**
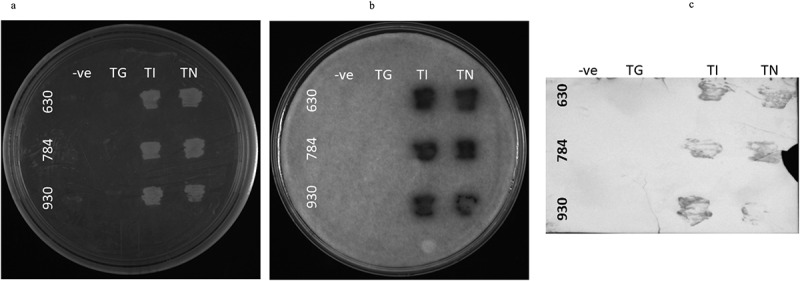
In vivo membrane-yeast-two-hybrid (MYTH) assay for OsGPCRLPs (630, 784 and 930) with RGA1. A. NMY51 yeast strain non-transformed negative control (TG), positive control (TI) and test (TN), plated on 4-dropout selection media supplemented with 0.5 mM 3-AT. B. Filter lift assay of plate a showing strength of interaction between high-ranked OsGPCRLP baits and RGA1 as prey. C. Western blot assay of plate a showing the specificity of the bait proteins.

All three OsGPCRLPs were cloned as bait proteins and assayed for functional expression and interaction with RGA1. Yeast cells co-transformed with the bait plasmid pTMBVα (930TMBV, 630TMBV, and 784TMBV) and prey plasmid (pPR3Nα-RGA1) showed prolonged growth of pinhead white to creamish colonies on 2D plates after one-week of incubation for all the selected vector combinations, indicating high transformation efficiency. Yeast cells expressing the positive controls (930TI, 630TI, and 784TI) and experimental constructs (930TN, 630TN, and 784TN) were positively selected on interaction selection media as evidenced by the robust growth of colonies on 3D and 4D selection plates (Figure 7a). The growth of yeast cells co-expressing 930TI, 630TI and 784TI confirmed the accurate expression, desired conformation and localization of the bait proteins. The appearance of indigo-colored bands on the BCIP-treated PVDF membrane in the case of all positive and positive test controls (TI and TN) further validated the expression of the functional bait proteins (Figure 7c). Substantial growth observed for 930TN, 630TN, and 784TN, suggest that positive bait-prey interactions led to reporter gene activation, thereby enabling the growth of host cells. No growth was observed in the negative controls, indicating appropriate selection of experimental controls. Subsequent growth of the selected 4D colonies on 4D +0.5 mM 3-AT supplemented media verified and eliminated the possibility of false positives. Furthermore, the filter-lift assay performed to assess the strength of the bait-prey interaction using X-gal, developed a blue color for the test (930TN, 784TN, and 630TN) and positive control samples (930TI, 784TI, and 630TI) only (Figure 7b).

## 4. Discussion

Plants have evolved sophisticated mechanisms to survive adverse conditions owing to their sessile habit. Membrane-localized heptahelical GPCRs and guanine nucleotide-mediated signal transduction are paramount mechanisms that enable plants to mitigate the temporal stresses and sustainably grow and develop. Metazoans have an extensive repertoire of GPCR:G protein components that plants lack. As GPCR is a vital signaling component in various metazoans, the search for GPCR analogs in plants is of primary significance and was accelerated after the characterization of GCR1 in Arabidopsis.^44^ Since then, several genome studies have identified potential GPCR-like proteins in plants.^9^^–41–69^ Crystallographic structures and MD simulation studies revealed significant similarities between plant Gαs, particularly AtGPA1, and the metazoan Gα_i_ family, with notable differences in the flexibility of the helical domain as well as inter- and intradomain interactions. The observed relaxation and dynamic mobility of the helical domain in comparison to the respective Ras-like domain endorse plant Gα with a GEF-independent nucleotide-exchange ability. This spontaneity causes GTP hydrolysis rather than GTP-binding, the rate-limiting step of the G protein cycle in plants.^70^ Phylogenetic studies advocate early eukaryotic origin and independent evolution of G proteins across eukaryotic lineages. This finding explains the differential receptor-G protein signaling architecture between opisthokonts and plants. While G proteins underwent greater expansion in metazoans, as evidenced by the multiplicity of heterotrimers, sequence divergence led to the origination of certain atypical Gαs in plants along with a finite set of canonical Gα, Gβ, and Gγ subunits. Presumably, these signaling elements enable plants to achieve the functional complexity integral to G protein signaling. Regardless, the functional dynamism of plant GPCR-G protein signaling is intriguingly comparable to that of animal systems. Maruta et al.^70^ also proposed the existence of a ternary complex, wherein membrane-bound RGS and heterotrimers are associated with single transmembrane receptor kinases rather than 7TM receptors, suggesting multisite phosphorylation-mediated co-regulation of the RGS protein and Gα in plants. Other nonconventional aspects of the heterotrimeric G protein cycle, as speculated by specific plant phenotypes, include invariable independence of XLGs, nucleotide exchange-independent canonical Gα functions, and conditional heterotrimer dissociation. Thus, in the case of plants, the interactome of the heterotrimeric complex is not restricted to 7TM receptors and RGS proteins but also encompasses distinct non-7TM receptors and effectors that seemingly regulate the G protein switch through an intricate posttranslational code.^71^ In contrast, another compendium of research substantiates the occurrence of a subset of 7TM receptors in addition to RGS proteins in plants. However, the plausible variations in the canonical theme might be due to the obvious differences in the biology of plants and animal systems. Despite the reviewed presence of archetype G protein-mediated signaling mechanisms, the existence of canonical GPCRs in plants is highly controversial.^25,44,67,72,73^

Since the first report of a plant GPCR in Arabidopsis (GCR1), there have been continuous attempts to validate the presence of GPCR-type 7TM receptors in the plant kingdom. Additionally, AtGCR1 is the first plant GPCR that has been validated for interaction with GPA1.^44^ However, the intrinsic GEF activity of AtGCR1 has yet to be elucidated. The computational study by Gookin et al.^67^ provides a pioneering approach to screening plant genomes for the identification of 7TM proteins with the typical architecture of a canonical GPCR using various bioinformatics platforms and prediction tools. Their study highlights the use of a bioinformatics pipeline exclusive to GPCR-mining in plants with sequenced genomes, and in vivo studies illustrated definite coupling between highly probable candidate GPCRs and AtGPA1. The sequence conservation of GPCRs is intrinsically as low as <25%, which depreciate the use of sequence homology-based identification of novel GPCRs.^45,74,75^

Invariably, GPCRs constitute the largest and most diverse family of type-I receptor proteins and have seven membrane-spanning helices with extracellular (ECL1–3) and intracellular loop (ICL1–3) regions. The laterally located N-terminus is free to interact with the diverse ligands, and the cytoplasm-located C-terminus interacts with the G protein heterotrimer (Gα, Gβ, and Gγ subunits) complex in a coupling-specificity-driven manner. The 3D spatial organization of intracellular regions along with the long C-terminal tail is highly dynamic and dictates the functional state of the receptor. Thus, topology prediction seems logical.

In the present study, we followed bioinformatics approaches to critically filter 7TM proteins from the rice proteome based on a topology prediction approach in the initial step in the quest for novel GPCR-like proteins using the 7TMRmine tool in combination with alignment-free and alignment-based algorithms with negligible disparities.^47^ The alignment-based methods recall proteins with significant similarity to known 7TMR subfamilies but fail to identify divergent 7TMRs. Such outliers can be sensitively identified using alignment-free methods. The combination of alignment-based and alignment-free methods facilitates the identification of a broader spectrum of 7TMR candidate proteins by complementing the limitations of each other. This integrated approach generated a pool of 25 proteins with putative 7TM topology (Table 3). Two-dimensional architecture and number of transmembrane domains on more stringent topology prediction platforms, *viz*. TMHMM2.0, SACSMEMSET, and TOPCONS sorted only three loci (LOC_Os06g09930.1, LOC_Os04g36630.1, and LOC_Os01g54784.1), out of 25 proteins, three have a typical GPCR-type ‘type-I’ heptahelical transmembrane topology, which in this study has been designated high-ranking candidate GPCR-like proteins. The idea behind the use of multiple tools for topological analysis based on complementary methods was to ensure the fidelity of the resulting data since the majority of such tools run on predictive models.

The overall results of superfamily, family and biologically significant sites prediction, as discussed in section 3.1.4, identified OsGPCRLP930 and OsGPCRLP784 as members of the established GPCR family based on conserved motifs. As per InterPro prediction, OsGPCRLP930 share similarity with the GCR1-cAMP receptor family (IPR022343: InterPro family) with the GCR1-cAMP receptor (PR02001: PRINTS) conserved motif and GPCR GCR1 family (IPR022340: InterPro family) with GCR1 (PR02000: PRINTS) signature motifs, while OsGPCRLP784 is a member of the THH1/TOM1/TOM3 family, which is also a part of the GPCR superfamily (Table 6).^76,77^ SUPERFAMILY2.0 shows relatedness of both OsGPCRLP930 and OsGPCRLP784 with the established Family A GPCR superfamily and the Rhodopsin-like family. OsGPCRLP630 belongs to an uncharacterized conserved protein family with currently no experimental data on the group or their homologs. Additionally, this group does not exhibit features indicative of any function (Interpro) and SUPERFAMILY 2.0 could not predict any family for this candidate (Table 6). All the posttranslational sites as enumerated by the PROSITE are typical of GPCRs and facilitate proper folding and localization. The presence of phosphorylation sites such as cAMP and cGMP-dependent phosphorylation sites, PKC, and CK II, confirms the occurrence of phosphorylation-mediated GPCR regulatory mechanisms.^78,79^

The presence of multiple protein kinase C phosphorylation sites and casein kinase II phosphorylation sites in OsGPCRLP930 and OsGPCRLP784 (Figure 4) seems to play key roles in the regulation of different cellular processes by inactivating GPCRs through feedback regulation by secondary messenger-activated kinases.^80^ Phosphorylation-mediated alterations in receptor conformation impair G protein heterotrimeric complex interactions. Such receptor regulation generally mediates ‘heterologous’ or nonagonist-specific deactivation. It occurs because stimuli that elevate cAMP (or diacylglycerol in the case of PKC) have the potential to phosphorylate and desensitize any GPCR containing a suitable PKC and/or PKA consensus phosphorylation site.^81^ The presence of casein kinase II phosphorylation sites reinforces the unidirectional desensitization of OsGPCRLPs after relaying the stimulus to downstream G protein-mediated signal transduction, as casein kinase II has been shown to phosphorylate substrates in hierarchical reactions.^82^ The presence of multiple *N-*myristoylation sites invariably aids in membrane anchoring,^83^ and multispanning of OsGPCRLPs might be involved in the differentiation of extracellular stimuli.

*N-*glycosylation, in general, facilitates the folding and trafficking of membrane proteins and is critical for GPCR-type specific functions.^84^ For example, the functions of the receptors rhodopsin,^85^ histamine H2,^86^ and angiotensin II type-1^87^ do not imply glycosylation. However, *N*-glycosylation reportedly has adaptable functions in the biosynthesis of β2 adrenergic receptors,^88^ the modulation of ligand binding affinity in vasopressin V1a receptors,^89^ and the regulation of G_*i*_-mediated signaling for P_2_Y_12_ receptors.^90^ In addition to affecting receptor functions, glycosylation also affects receptor expression levels of some GPCRs, including rhodopsin, β2 adrenergic, angiotensin II type-1, and orphan Gpr176.^84^ The OsGPCRLP930 has two *N-*glycosylation sites, whereas OsGPCRLP784 and OsGPCRLP630 lack glycosylation sites. Presence of a leucine zipper motif which is required for dimerization, in the OsGPCRLP630 suggests its possible role in homo or heterodimerization.^91^

In addition to the 7TM scaffold, highly conserved consensus motifs and signature salt and disulfide bridges dispersed across various transmembrane helices and intracellular regions that precisely create GPCR-specific folds are other features of its structure. Fold recognition studies on AtGCR1 using class A, B, and F GPCRs as templates revealed that AtGCR1 has a GPCR fold that, more than any other observation (lack of GEF activity), verifies its status as a plant GPCR.^72^ The presence of definitive GPCR-fold specific residues and motifs in our high-ranked OsGPCRLPs strengthens their potential as true GPCR manifolds. Given that, RGS-encoding genes have been lost from the grass family owing to the Thr→Asn substitution in Gα in most monocots,^92^ the sequence alignment of putative high-ranked OSGPCRLPs with known human and Arabidopsis RGS proteins revealed no significant similarities (Figure 6). Thus, the absence of the C-terminal RGS box domain in the proposed putative OsGPCRLPs indicate that these 7TMR proteins are not members of the 7TM-RGS protein family. Moreover, the same sequence alignment highlights the significant similarity in OsGPCRLP930, which strongly suggests that rice proteome has GPCRLPs similar to Arabidopsis GCR1 and lotus GPCRs.^44,61^ Multiple sequence alignment of the OsGPCRLP630 and OsGPCRLP784 annotated them as RGS domain-lacking 7TM proteins. Thus, all this rationalized evidence strongly indicates that the identified high-ranked OsGPCRLPs might be relatives and new members of the limited plant GPCR repertoire.

Pertinent evidence suggests that the identified OsGPCRLPs might be the members of noncanonical GPCR families. Analyses of their probable molecular and biological functions and subcellular localization and gene ontology annotations indicated their active role in cell communication and potential association with cell surface receptor signaling pathways. Notably, the OsGPCRLP930 and OsGPCRLP784 were identified as integral components of GPCR signaling pathways. However, GPCR signaling-oriented functions were not directly predicted in the case of OsGPCRLP630 (Table 6).

Moreover, a 7TM protein may function as a canonical GPCR if it possesses the intrinsic ability to induce conformation-driven nucleotide exchange in the Gα subunit of the heterotrimeric G protein complex. In pursuit of this feature in our high-ranked GPCRLPs, our analysis of the STRING database showed that all three OsGPCRLPs interacted with all typical GPCR-interacting proteins (Figure 5). An interactome map of OsGPCRLP930 revealed that it interacts with a putative XLG (STRING: OsJ_19863) along with the classical G protein subunits D1, RGB1, RGG1 and RGG2 (Figure 5a). Additionally, the OsGPCRLP784 and OsGPCRLP630 were shown to interact with typical heterotrimeric components with the additional interacting partner GPCR-type G protein COLD1 (Figure 5b,c, respectively). Interestingly, COLD1 is a plasma membrane and endoplasmic reticulum-localized regulator of G protein signaling (RGS) that bestows low temperature tolerance in rice^42,69,93^ and was reported to be a functional substitute for the classical RGS proteins.^94^ However, the interactome map of the OsGPCRLP630 was variable at medium confidence, and RGG2 was not mapped in the interaction network. Moreover, two unknown kinase domain-containing proteins were identified. This finding likely indicate unique phosphorylation-dependent function of the OsGPCRLP630.

The interaction of Gα with GPCRs is not random but is specified by the dynamic spatial organization of the intracellular domains of GPCRs, which are known to have intrinsic GEF activity. The coupling specificity of GPCR and Gα thus provides selectivity for downstream effectors and signaling routes to be activated. Plant Gα subunits are the closest homologs of the G_i_-type Gα family. Generally, GPCRs function in a Gα-dependent manner; that is, a given GPCR is more likely to interact with and activate a single subtype of the G protein family. Nonetheless, this specificity is not uniform, as numerous GPCRs have been shown to couple with more than one Gα-type in animals.^95^ The absence of sequence homology and consensus sequence motifs for G protein coupling in GPCRs associated with the same G protein subtype suitably supports the fact that, upon binding of a ligand, the 3D conformation switching by the three intracellular loops along with the C-terminal tail of GPCRs governs G protein selectivity. These transitions, when propagated to the flexible switch regions in Gα, facilitate the nucleotide exchange-triggered dissociation of the Gβγ heterodimer.^4^ Studies on GPCR:G protein selectivity suggest that a large subset of nonolfactory GPCRs exhibit selectivity for G_i/o_-type Gα (43%), followed by 33% of G_q/11_-type and 25% of G_s_-coupled GPCRs.^95^ We observed a similar tendency of Gα coupling specificity for the OsGPCRLP930 and OsGPCRLP784, with the highest coupling specificity for G_i/o_-type Gα, followed by G_q/11_. However, unlike that of OsGPCRLP930, the coupling specificity of OsGPCRLP784 with G_s_ is negligible. Moreover, OsGPCRLP630 exhibited the highest Gα-selectivity for G_q/11_-type (Table 7). The search for animal orthologs of candidate OsGPCRLPs also strongly supported that the identified OsGPCRLPs are strongly related to well-known animal GPCR families (Table 8).

Affirmative indications from the computational analysis were strongly supported by the in vivo coupling assay between high-ranked OsGPCRLPs and RGA1 using the MYTH assay exclusively modified for membrane proteins. The prolific growth of yeast cells coexpressing the OsGPCRLPs and RGA1 under stringent levels of auxotrophic selection, 4D – SD agar supplemented with 0.5 mM 3-AT, strongly indicates a positive and strong interaction between the OsGPCRLPs expressed in the native conformation and native RGA1 (Figure 7).

In conclusion, rigorous bioinformatics analysis and strong positive interactions between the identified subset of OsGPCRLPs and RGA1 suggest that the rice genome encodes a subset of 7TM receptor-like proteins that are structurally and functionally at par with canonical GPCRs. It also provides promising information regarding the extension of the GPCR-mediated signaling repertoire in plants. However, by observing the existing evidences and self-activation paradox of Gα, the possible intrinsic GEF activities of these novel rice GPCRLPs and their roles in the biochemical mechanism of the RGA1 activation/deactivation cycle can be examined.

## Supplementary Material

Supplementary Tables.xlsx
